# Maternal Fatty Acid Metabolism in Pregnancy and Its Consequences in the Feto-Placental Development

**DOI:** 10.3389/fphys.2021.787848

**Published:** 2022-01-20

**Authors:** Asim K. Duttaroy, Sanjay Basak

**Affiliations:** ^1^Department of Nutrition, Faculty of Medicine, Institute of Basic Medical Sciences, University of Oslo, Oslo, Norway; ^2^Molecular Biology Division, ICMR-National Institute of Nutrition, Indian Council of Medical Research, Hyderabad, India

**Keywords:** arachidonic acid, docosahexaenoic acid, placenta, fetus, FATP, FABP, angiogenesis, fetal development

## Abstract

During pregnancy, maternal plasma fatty acids are critically required for cell growth and development, cell signaling, and the development of critical structural and functional aspects of the feto-placental unit. In addition, the fatty acids modulate the early stages of placental development by regulating angiogenesis in the first-trimester human placenta. Preferential transport of maternal plasma long-chain polyunsaturated fatty acids during the third trimester is critical for optimal fetal brain development. Maternal status such as obesity, diabetes, and dietary intakes may affect the functional changes in lipid metabolic processes in maternal-fetal lipid transport and metabolism. Fatty acids traverse the placental membranes *via* several plasma membrane fatty acid transport/binding proteins (FAT, FATP, p-FABPpm, and FFARs) and cytoplasmic fatty acid-binding proteins (FABPs). This review discusses the maternal metabolism of fatty acids and their effects on early placentation, placental fatty acid transport and metabolism, and their roles in feto-placental growth and development. The review also highlights how maternal fat metabolism modulates lipid processing, including transportation, esterification, and oxidation of fatty acids.

## Introduction

**FIGURE 1:**
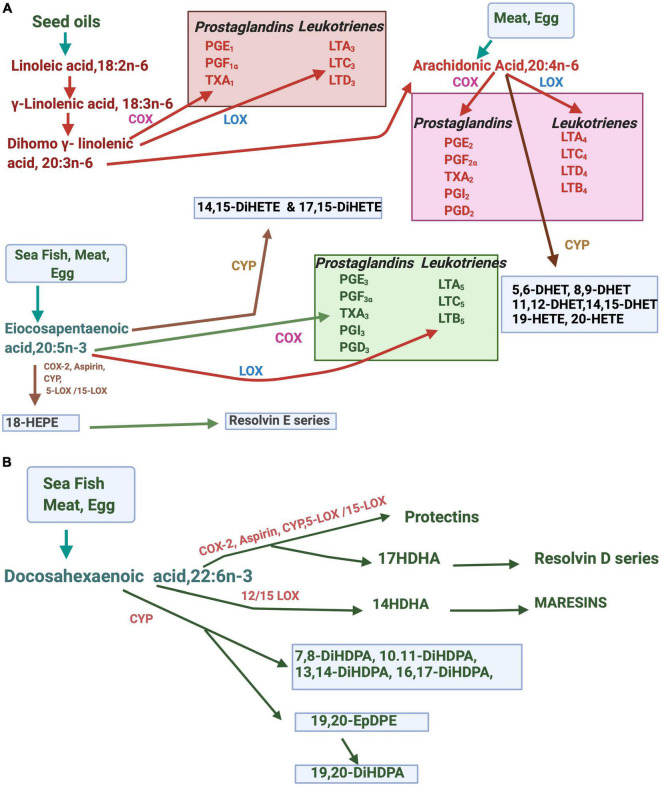
Metabolites of EPA, DHA, and ARA in the body. **(A)** Arachidonic acid (ARA), and eicosapentaenoic acid (EPA) are metabolized by enzymes such as cytochrome P450 (CYP), lipoxygenase (LOX), or cyclooxygenase (COX). **(B)** Metabolism of docosahexaenoic acid (DHA) to docosanoids and other derivatives. DHET, dihydroxyeicosatrienoic acids; HETE, hydroxyeicosatetraenoic acid; diHETE, dihydroxy eicosatetraenoic acid, PG, prostaglandin: LT, leukotrienes; diHDPA; dihydroxydocosapentaenoic acid.

Fatty acids have vital functions on energy metabolism and storage as they participate in cell enlargement, promote cell functions, regulate gene expression, coordinate intra- and extracellular communications, regulate the supply of energy substrates, and control cellular responses to the metabolic environment (Crabtree et al., 1998; Mallick et al., 2021). Fatty acids modulate membrane architecture, dynamics, and functionalities (Brash, 2001; Nakamura et al., 2001). Besides, they are functionally involved in regulating membrane ion channels and receptors and cell signaling (Kang and Leaf, 1996; Kim and Pleumsamran, 2000; Bazan, 2003; Carattino et al., 2003; Danthi et al., 2005). Linoleic acid, 18:2n-6 (LA), and alpha-linolenic acid 18:3n-3 (ALA) are the two essential fatty acids (EFAs) that belong to the n-6 and n-3 families of fatty acids. Both n-3 and n-6 fatty acids and their long-chain polyunsaturated fatty acids (LCPUFAs) play critical roles that include the structure and function of cell membranes as signaling molecules, act ligands for gene expression and maintain cellular metabolic homeostasis (Smith, 1989; Innis, 1991; Dutta-Roy, 1994). The metabolism of n-6 and n-3 EFAs is tightly regulated by the interplay of several enzymes such as desaturases, elongases, cyclooxygenases (COXs), lipooxygenases (LOXs), and cytochrome P450 system. N-3 and n-6 fatty acids metabolically produce compounds with diverse physiological and pathological functions. Eicosanoids are synthesized from n-3, and n-6 LCPUFAs include prostaglandins (PGs), thromboxanes (TXs), leukotrienes (LTs), and hydroxyeicosatetraenoic acids (HETEs). The source and metabolism of LCFAs and their various metabolites are summarized in Figures 1A,B. ARA, dihomo-γ-linolenic, or 20:3(*n*-6), EPA, and DHA are obtained from the diet or produced in the body from these EFA precursors. Multiple stimuli lead to the release of membrane-bound LCPUFAs such as ARA, dihomo-γ-linolenic acid,20:3*n*-6 (DGLA), EPA, and DHA *via* activation of cellular phospholipases. Cycloxygenase (COX) and lipoxygenase (LOXs) are involved in producing LCPUFA-metabolites, eicosanoids. Several LOXs act upon ARA, EPA, DGLA, and DHA positions. The docosanoids, such as protectins, resolvins, and maresins or “specialized pro-resolving mediators,” are derived from DHA. E-series resolvins are produced from EPA. The cytochrome P-450 epoxygenase pathway produces hydroxyeicosatetraenoic acids (HETEs) and epoxides (EETs). While many of the requisite enzymes, precursors, and products are specific to particular types of cells, the proximity of some cell types can facilitate the transfer of eicosanoids between cells for further metabolism, and for example, some of the leukotrienes are produced by trans-cellular mechanisms.

Eicosanoids are involved in controlling various physiological and pathological processes in the body. Eicosanoids produced from n-6 LCPUFAs promote inflammation, tumor growth, and angiogenesis (Smith, 1989; Nie et al., 2000; Hoagland et al., 2001; Pai et al., 2001; Bagga et al., 2003; Pola et al., 2004; Kamiyama et al., 2006; Jin et al., 2009). In contrast, n-3 derived eicosanoids usually have anti-inflammatory, anti-cancer, and anti-angiogenic properties. LCPUFAs such as arachidonic acid,20:4n-6 (ARA) and docosahexaenoic acid,22:6n-3 (DHA) are involved in the development of the central nervous system, visual acuity, and cognitive functions (Budowski and Crawford, 1985; Innis, 1991; Jeffrey et al., 2001; Uauy et al., 2001). These fatty acids also regulate the expression of genes involved in fatty acids biosynthesis and oxidation, lipogenesis, glucose utilization, and insulin sensitivity, thermoregulation, energy partitioning, cholesterol transport, cholesterol synthesis, and cell growth and differentiation (Long and Pekala, 1996; Jump, 2002; Wahle et al., 2003; Wilke and Clandinin, 2005; Mallick and Duttaroy, 2021).

**FIGURE 2:**
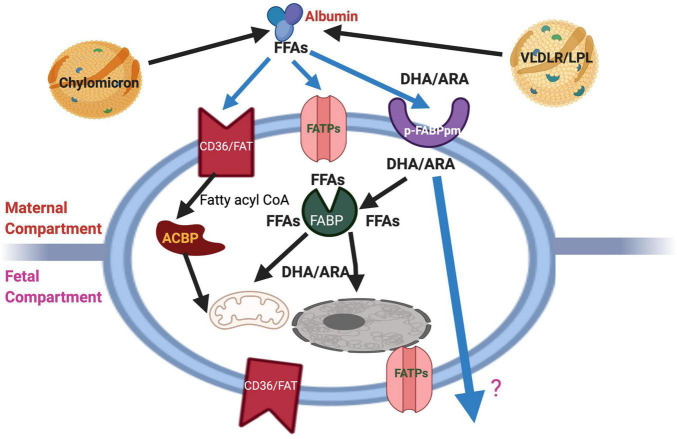
Schematic diagram showing the cellular proteins and their involvement in the transport of maternal fatty acids across the human placental trophoblast. The schematic overview of proteins involved in fatty acid uptake and transport in last trimester trophoblast cells. The location of FAT and FATP on both sides of the bipolar placental cells and the lack of specificity for particular types of FFAs allow transport by all fatty acids bi-directionally, i.e., from the mother to the fetus and vice versa. However, by virtue of its exclusive location on both sides and preference for ARA and DHA, p-FABPpm is thought to be involved in sequestering these maternal plasma LCPUFAs to the placenta. In addition, cytoplasmic FABPs may be responsible for the trans cytoplasmic movement of FFAs to their sites of esterification, β-oxidation, or fetal circulation *via* placental basal membranes. FATP, Fatty acid transporter protein; pFABPpm, Plasma membrane fatty acid-binding protein; ACBP, Acyl-CoA binding protein.

The fundamental roles of fatty acids as structural components and functional modulators (Duttaroy and Basak, 2020) define maternal metabolism of fatty acids is an essential factor for feto-placental development. The brain and the retina are rich in LCPUFAs such as ARA and DHA. An abundant supply of these LCPUFAs during pregnancy and the neonatal period is critical to ensure optimal brain development and functionalities (Basak et al., 2020; Dutta-Roy, 2000b). Pre-term babies have significantly lower EFA and LCPUFAs statuses than do full-term neonates. The dietary EFA deficiency has become a pertinent issue for pre-term infants’ nutrition because they do not receive the third-trimester intrauterine supply of DHA and ARA. Supplementation of pregnant mothers with LCPUFAs has improved neonatal LCPUFAs status (Judge, 2018; Middleton et al., 2018). It is concluded that LCPUFAs levels are higher in fetal than in maternal circulation *via* different mechanisms such as maternal lipid metabolism and preferential placental transport (Zamai et al., 2020; Basak et al., 2021). Several proteins such as cytoplasmic fatty acid-binding proteins (FABPs), plasma membrane fatty acid-binding protein (FABPpm), fatty acid translocase (FAT/CD36), fatty acid transporter proteins (FATPs), and free fatty acid receptors (FFARs) bind FFAs, whenever these are available in the extracellular medium, the cytosol, or the nuclear matrix (Duttaroy, 2009; Hara et al., 2013; Mallick et al., 2021). FFAs are taken up by cells by their membrane transport/binding proteins such as FABPpm, FAT/CD36, and FATPs. At the plasma membrane, FFAs can also activate FFARs, known as G protein-coupled receptors (GPRs) (Hara et al., 2013) while they bind FABPs in the cytosolic compartment and are processed to subcellular structures or metabolic pathways. Another essential lipid metabolic regulator is peroxisome proliferator-activated receptors (PPARs), ligand-activated transcription factors, which mediate the regulation of fatty acids in the nucleus (Zolezzi and Inestrosa, 2013). Currently, the regulatory and signaling roles of FFAs are gaining importance in physiological and pathological processes as these receptors are better characterized (Mallick et al., 2021). Figure 2 describes the schematic diagram showing proteins involved in transporting maternal fatty acids across the human placental trophoblast.

This review brings together recent developments on the metabolism of fatty acids in mothers and their impacts on fetoplacental development.

## Maternal Lipid Metabolism Affects Pregnancy

Pregnancy is generally marked with hyperlipidemia involving all lipid classes such as lipoproteins, triacylglycerols (TAGs), and free fatty acids (FFAs) (Punnonen, 1977; Darmady and Postle, 1982; Jimenez et al., 1988). The elevated lipid levels facilitate fetal access to the available fatty acids (Warth et al., 1975). The plasma levels of phospholipids increase by approximately 65% during the third trimester as compared first trimester of pregnancy (Punnonen, 1977). The fat deposition in mothers is primarily aimed to meet the lipid demand of the developing fetus. Maternal hyperlipidemia during late pregnancy plays a crucial role in maternal metabolic adaptations that are geared to benefit fetal growth. The fat mass in adipose tissue increases during pregnancy, even in malnourished mothers (Prentice and Goldberg, 2000; Herrera, 2002; Herrera et al., 2006). During fat deposition, the levels of different lipid fractions in plasma, such as lipoproteins, TAGs, phospholipids, FFAs, are increased in maternal circulation. Lowered insulin-dependent decrease in lipoprotein lipase (LPL) activity in adipose tissue and subsequent insulin resistance are observed during the pregnancy. Maternal lipolysis during gestation is regulated by insulin, glucose, progesterone, cortisol, prolactin, estrogen, adipokine, and leptin (Cousins, 1991; Catov et al., 2007). They inhibit hepatic LPL activity while increasing intestinal absorption of dietary fats (Cetin et al., 2009). These dynamic physiological changes increase the levels of circulating FFAs and glycerol, which are substrates for hepatic synthesis of very-low-density lipoproteins (VLDL). Adipose mass and hyperlipidemia are elevated in pregnant mothers, while the requirement of LCPUFAs is accelerated for fetal growth and development. The rapid increase in adipose mass is the result of both hyperphagia (Murphy and Abrams, 1993) and increased lipid synthesis driven by the enhanced adipose tissue insulin sensitivity during early pregnancy (Ramos et al., 2003). During early pregnancy, there is unchanged or even increased adipose tissue LPL activity. The LPL enzyme is extrahepatic, mainly active in adipose tissue, and catalyzes the hydrolysis of circulating TAGs in TAG-rich lipoproteins such as chylomicrons and VLDLs (Mead et al., 2002). The hydrolytic products, FFAs and 2-monoacylglycerol or glycerol, are taken up by the subjacent tissue for re-synthesis of TAGs. These changes facilitate the accumulation of circulating lipids in maternal fat depots. However, the increased accumulation of maternal adipose depot stops during the third trimester of pregnancy due to decreased adipose fatty acid synthesis (Ramos et al., 2003), and reduced LPL activity. The reduced activity of these enzymes causes a reduction in the hydrolysis and tissue uptake of TAGs in TAG-rich lipoproteins and contributes to the development of maternal hypertriglyceridemia; and increased adipose lipolytic activity (Elliott, 1975). The insulin resistance in the last trimester of pregnancy (Dahlgren, 2006; Barbour et al., 2007) contributes to these changes (Sivan et al., 1999). Pregnancy-specific hormones have insulin-antagonistic and lipolytic effects (Ryan and Enns, 1988). Increased blood levels of human placental lactogen, placentally derived human growth hormone, human chorionic gonadotropin, progesterone, cortisol, prolactin, and other hormones increase insulin resistance in peripheral tissues such as adipocytes and skeletal muscle (Newbern and Freemark, 2011). Increased levels of progesterone, cortisol and other hormones markedly decreased insulin sensitivity during the second trimester onward, with the highest insulin resistance noted at third trimester (Di Cianni et al., 2003). In addition, changes in the production of inflammatory mediators by the placenta and cytokines produced by adipose decrease insulin sensitivity (Di Cianni et al., 2003; Desoye and Hauguel-De Mouzon, 2007; Lain and Catalano, 2007). Central leptin resistance leads to an increased body weight despite the catabolic state in late gestation. During early pregnancy, maternal adipose fat accumulation may represent a store of essential lipid components derived from the maternal diet.

At first, TAG in the form of both liver-derived VLDL and chylomicrons must be hydrolyzed to FFA for placental supply to the fetus. The two primary human placental lipases, placental lipoprotein lipase and endothelial lipase release FFAs from lipoproteins. In maternal adipose, LPL is the major TAG hydrolase, and it likely plays a significant role in the placenta (Waterman et al., 1998, 2000; Magnusson-Olsson et al., 2006). Adipose lipoprotein lipase hydrolyses TAGs from VLDL and chylomicrons to FFA for adipose’s lipid uptake in the early stage of pregnancy when mothers are relatively insulin sensitive. However, during late gestation specific insulin resistance, activity reduces to support maternal energy requirements while glucose is diverted to the growing fetus (Kim et al., 2014; Herrera and Desoye, 2016). In the placenta, hydrolysis of TAG to FFA by placental lipases allows them to be taken up by the syncytiotrophoblast, where they can be metabolized and transported into fetal circulation (Waterman et al., 2000; Barrett et al., 2014; Heerwagen et al., 2018). As the liver is the site of LCPUFA synthesis, it is suggested that VLDL may play an important role in transporting plasma LCPUFAs in pregnancy. However, a ratio of HDL lipoprotein of ARA and DHA to HDL cholesterol and HDL- apoAI, enrichment of plasma HDL with ARA and DHA, suggest DHA is primarily carried by HDL during pregnancy (Zamai et al., 2020). Several mechanisms ensure adequate transport of these LCPUFAs to the fetus (Duttaroy, 2009). The fetal brain has its growth spurt in the third trimester of pregnancy and early childhood. The central nervous system is particularly rich in ARA and DHA, and the cerebral accretion of these LCPUFAs is essential (McNamara, 2010; Parellada et al., 2017; Basak et al., 2021). Therefore, an appropriate pre-and postnatal supply of these LCPUFAs is necessary for optimal fetal and neonatal growth, neurologic development and function (Innis, 1991; Makrides et al., 2001), learning and behavior (Stevens et al., 1995; Basak et al., 2021).

The plasma levels of phospholipid-containing ARA and DHA increase on average by ∼40% from the first trimester (10 weeks) to the time of parturition. However, non-essential unsaturated fatty acids increase considerably with the progression of gestation (>65%). Therefore, maternal EFA supply is continuously required for fetal growth and development, as the fetus depends on the maternal supply of LCPUFAs such as ARA, EPA, and DHA. A deficiency of these essential fatty acids may critically affect embryonic organogenesis, particularly in neurological development. LCPUFAs derived from the diet are stored in the maternal adipose depot during early pregnancy. The maternal body fat accumulation allows an essential store of LCPUFAs derived from the maternal diet and metabolism. Since the conversion of EFAs to DHA and ARA is relatively low in humans, maternal availability and supply of LCPUFA are essential for the developing fetus. Thus, the diet of pregnant women must contain sufficient LCPUFAs to meet the metabolic requirements of the mother and the developing fetus.

## Long-Chain Fatty Acids and Early Placentation Processes

Early placentation requires the invasive properties of the extravillous trophoblasts derived from the outer trophectoderm layer of the blastocysts (Moser and Huppertz, 2017). Insufficient trophoblast invasion of the uterine wall and resultant reduced blood flow can complicate the pregnancy outcome, as observed in preeclampsia. Therefore, invasive extravillous trophoblasts of the human placenta are critically factored in a successful pregnancy. These trophoblasts remodel the uterine spiral arteries to increase blood flow and oxygen delivery to the developing fetus. This invasive behavior of trophoblasts follows a precise chronology of vascular events during early gestation. The development of a placental vascular network is essential for the growth and maintenance of the developing fetus (Chung et al., 2000; Chaiworapongsa et al., 2011; Basak et al., 2013). Defective invasion of trophoblasts in the uterine wall is directly involved in the development of preeclampsia, a major and frequent complication of human pregnancy with detrimental consequences on fetal and maternal health. The trophoblastic invasion of the uterine wall is precisely regulated. It is transiently restricted to early pregnancy, and it is spatially confined to the endometrium, the first-third of the myometrium, and the associated spiral arteries. The remodeling of spiral arteries mimics the process of angiogenesis and is critical for the early placentation process. The angiogenesis process involves several factors such as vascular endothelial growth factor (VEGF), angiopoietin-like protein 4 (ANGPTL4), platelet-derived growth factor (PDGF), and platelet-activating factor (PAF) (Chung et al., 2000; Hill, 2001; Chaiworapongsa et al., 2011; Basak et al., 2013). EFAs and LCPUFAs metabolites modulate angiogenesis processes (Hardman, 2004; Spencer et al., 2009; Salvado et al., 2012). Eicosanoids produced from ARA stimulate angiogenesis, whereas those produced from EPA and DHA inhibit angiogenesis and tumorigenesis (Hardman, 2004; Spencer et al., 2009; Salvado et al., 2012). Both n-3 and n-6 LCPUFAs and their eicosanoids regulate several angiogenic growth factors, cell migration, proliferation, and angiogenesis. N-3 fatty acids and their eicosanoid derivatives attenuate excess vascularization by reducing angiogenesis through decreased production of proangiogenic ARA-derived eicosanoids, membrane receptor-ligand interactions, and *via* their intrinsic anti-tumor activities (Nie et al., 2000; Pai et al., 2001; Bagga et al., 2003; Pola et al., 2004; Kamiyama et al., 2006). However, the angiogenic response is more complex and may involve several other factors (Dimitriadis et al., 2005; Spencer et al., 2009). Several n-6 eicosanoids promote tumor growth and angiogenesis. PGE_2_, produced from ARA, can induce angiogenesis by producing various proangiogenic factors in different cells (Spencer et al., 2009; Tobin et al., 2009; Zhang and Daaka, 2011; Basak et al., 2013). PGE_2_ increased the synthesis of VEGF, basic fibroblast growth factor (bFGF), or CXCL1 that, in turn, act on endothelial cells to promote angiogenesis (Weedon-Fekjaer et al., 2005; Wang et al., 2006; Spencer et al., 2009; Zhang and Daaka, 2011). The PGE_2_-induced angiogenic response is mediated *via* PGE_2_ receptor-mediated signaling systems. Since PGE_2_ is produced from ARA by COX-2, thus COX-2 is regarded as an angiogenesis mediator. Overexpression of COX-2 is significantly correlated to tumor invasiveness, prognosis, and survival in some cancers (Fu et al., 2004). Thus, COX-2 inhibitors can effectively prevent n-6 eicosanoids mediated inflammation, proliferation, angiogenesis, and apoptosis (Fu et al., 2004). In contrast, n-3 fatty acids reduce the expression of VEGF, PDGF, IL-6, and MMP-2 (Kang and Weylandt, 2008; Spencer et al., 2009). In marked contrast to the effects observed with the n-3 LCPUFAs, the n-6 LCPUFAs have a stimulatory or neutral effect on angiogenic processes, cell migration, or cell proliferation (Hardman, 2004; Szymczak et al., 2008). During the initiation and progression of tumors, several enzymes involved in the biosynthesis of eicosanoids, such as COX-2, 5-LOX, and 12-LOX, are upregulated. Overexpression of COX-2 in carcinoma cells increases angiogenesis, as evidenced by an increased migration and tube formation of the endothelial cells *via* producing eicosanoids and angiogenic factors, VEGF and bFGF. Besides, inflammatory cells in the tumors may release pro-inflammatory cytokines such as IL1β or TNFα that can induce angiogenesis. The anti-angiogenic activity of n-3 LCPUFAs is mediated by inhibiting the synthesis of ARA-derived eicosanoids (Spencer et al., 2009). VEGF or its receptors are upregulated in several tumors (Takahashi, 2011). N-3 fatty acids affect the expression of proangiogenic factors (Massaro et al., 2010; Scoditti et al., 2010). Both EPA and DHA significantly inhibit cell proliferation, migration, and tubule formation in endothelial cells (Kanayasu et al., 1991; Yang et al., 1998; Murota et al., 2000; Kim et al., 2005; Spencer et al., 2009). VEGF receptors are down-regulated by EPA by inhibiting activation of NFκB (Ghosh-Choudhury et al., 2009; Spencer et al., 2009). Moreover, EPA down-regulates Flk-1 receptors expression, whereas it upregulates the expression of Flt-1 receptors (Yang et al., 1998). N-3 LCPUFAs suppressed the VEGF-induced tube formation *via* down-regulation of VEGFR-2 in the endothelial cells (Tsuji et al., 2003; Calviello et al., 2004; Szymczak et al., 2008; Spencer et al., 2009). EPA and DHA reduce angiogenesis in tumors by inhibiting the synthesis of angiogenic factors such as VEGF, PDGF, COX-2, PGE_2_, nitric oxide (NO) (Spencer et al., 2009). 4-hydroxy-DHA, a 5-Lipoxygenase product of DHA, inhibits endothelial cell proliferation and angiogenesis mediated *via* PPARγ (Abdelwahab et al., 2003; Sapieha et al., 2011). Increased NO levels by n-3 fatty acids may decrease VEGFR-mediated angiogenesis (Matesanz et al., 2010). A higher ratio of O_2_^–^/NO contribute to disturbing angiogenesis (Matesanz et al., 2010; Hamed et al., 2011).

Since n-6 fatty acids are involved in tumor progression and metastasis *via* stimulated angiogenesis, reducing the ratio of n-6/n-3 fatty acids in tissue may impart benefit in cancers (Clarke, 2000; Price et al., 2000; Nakamura et al., 2001; Wahle et al., 2003). N-3 LCPUFAs reduce angiogenesis *via* several mechanisms, including modulation in the expression of VEGF, ANGPTL4, eicosanoids, COX-2, fatty acid-binding proteins (FABPs), and nitric oxide (NO) (Spencer et al., 2009). DHA, in contrast, increased tube formation to the greatest extent compared with EPA and ARA in extravillous trophoblasts, HTR8/SVneo cells. Table 1 shows the effects of fatty acids on the expression of mRNAs of angiogenic growth factors in these cells. DHA stimulated tube formation by stimulating the expression and secretion of VEGF in extravillous trophoblast cells (Johnsen et al., 2011). Thus, DHA can help the early placentation process by stimulating angiogenesis. This contrasts with the inhibitory effects of DHA on angiogenesis in many cell types, including tumors (Spencer et al., 2009). The mRNA expression of VEGF by DHA is unique as various growth factors and cytokines induce it but not by any other fatty acids. However, DHA stimulates VEGF expression and secretion, whereas all long-chain fatty acids stimulate ANGPTL4 secretion. In addition to stimulating the expression of major angiogenic factors such as VEGF and ANGPTL4, fatty acids also stimulate the expression of intracellular FABP-4 and FABP-3, which are known to modulate angiogenesis directly. Emerging data indicate that FABPs may be involved in the angiogenesis process. This indicates that differential mode of action of DHA compared with other fatty acids in the angiogenic process (Johnsen et al., 2011). Effects of these fatty acids were quite the opposite to what was observed in tumors (Basak et al., 2013; Kang and Liu, 2013). With the recent spate of studies on regulators of angiogenesis, these observations on the regulation of placental angiogenesis could be exploited for ensuring positive outcomes for most pregnancies.

**TABLE 1:** Shows the effects of fatty acids on the expression of mRNAs of angiogenic growth factors in these cells.

Parameters	Fold change of mRNA normalized with TBP	
**Fatty acids (100 μM)**	**VEGF**	**ANGPTL4**
Control	1.0	1.0
OA	0.96	8.5
t-9 ELA	0.76	1.95
AA	1.2	18*
c9,t11-CLA	1.13	12.43*
EPA	1.9	20.0*
DHA	2.1*	23.7*

*p < 0.05 vs. control.

*Reprinted from Life Sciences, 93, 755-762, 2013. Fatty acid-induced angiogenesis in first-trimester placental trophoblast cells: Possible roles of cellular fatty acid-binding proteins, With permission from Elsevier.*

FABPs have a role in angiogenesis, and their expression is modulated by various angiogenic mediators (Mousiolis et al., 2012). Both VEGF and bFGF increase the mRNA and protein levels of FABP4 in endothelial cells. FABP4 promotes the proliferation of endothelial cells. First-trimester trophoblast cells express and secrete VEGF considerably in the presence of fatty acids and FABP4 *via* VEGF, ANGPTL4, and FABP4. FABP4-induced VEGF expression is inhibited by siRNA-mediated knockdown of VEGFR2, whereas the VEGFR1 agonists, PIGF1 and 2, had no such effects. FABP4 is a novel target of the VEGF/VEGFR2 pathway and acts as a positive regulator of angiogenesis in endothelial cells (Elmasri et al., 2009, 2012; Cataltepe et al., 2011; Ghelfi et al., 2013). Expression of FABP4 is demonstrated in an angiogenesis-dependent pathology, infantile hemangioma, the most common tumor of infancy, and endothelial cells (Fishman and Mulliken, 1993). FABP4 has a role in activating mitogenic pathways and the expression of several key mediators of angiogenesis. Leptin, EPA, and DHA- stimulated FABP4 expression possibly target VEGF signaling in trophoblast HTR8/SVneo cells (Basak and Duttaroy, 2012; Basak et al., 2013).

**FIGURE 3:**
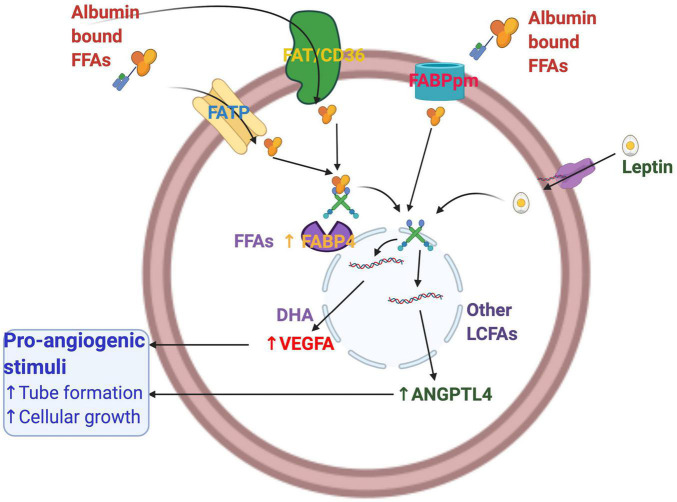
Fatty acid-induced angiogenesis in the human placental first-trimester trophoblasts. Proangiogenic growth factors such as ANGPTL4, VEGF, and FABP4 are involved in placental angiogenesis. ANGPTL4 and FABP4 are stimulated by all other long-chain fatty acids, whereas DHA stimulates VEGF. DHA, Docosahexaenoic acid; FABP4, Fatty acid-binding protein 4; VEGF, vascular endothelial growth factor; ANGPTL4, angiopoietin-like protein 4; LCFAs, long-chain fatty acids.

Despite the similarity in ligand binding property and tertiary structures, FABPs have a tissue-specific function (Storch and Thumser, 2010). Maternal plasma FABP4 is independently related to the development of preeclampsia (Yan et al., 2016). FABP4 involves preeclampsia’s pathogenesis *via* different pathways associated with insulin resistance, inflammation, and dyslipidemia. Increasing evidence suggests the angiogenic function of fatty acid transport/binding proteins in various cell systems (Akbal et al., 2013; Ghelfi et al., 2013). In placental trophoblasts, angiogenesis was maximally blocked in the presence of FABP4 inhibitor compared with other VEGF signaling inhibitors such as P38/MAPK, L-NAME. However, DHA- and VEGF- induced tube formation was maximally reduced by p38 MAP kinase inhibitor. All these inhibitors could not inhibit the ANGPTL4 and oleic acid-induced tube formation in placental trophoblasts. FABP4, therefore, may be involved in part in the basal level and stimulated tube formation by VEGF, DHA, and leptin, whereas it has little or no effect in ANGPTL4- and OA-induced tube formation in these cells (Pandya et al., 2016). FABP4 may play a differential role in fatty acids, and angiogenic growth factors mediated tube formation in the first-trimester trophoblast cells. Figure 3 describes the fatty acid-induced angiogenesis in the human placental first-trimester trophoblasts.

## The Human Placental Fatty Acid Transport System: An Overview

Fatty acids provide cellular energy, constitute an essential structural element of cellular membranes, and are important for developing specific tissues. The fetal source of fatty acids is either TAG-rich maternal lipoproteins, such as chylomicrons and VLDL, or FFAs bound to albumin. Albumin–FFAs complexes interact with fatty acid uptake/transport proteins in the microvillous plasma membrane (MVM), taking FFAs into the syncytiotrophoblast cells (Duttaroy, 2009). TAG in maternal lipoproteins, particularly very-low-density lipoproteins (VLDL), is hydrolyzed into FFAs by lipoprotein lipase (LPL) expressed in the MVM. FFA is subsequently transferred across the MVM. Alternatively, maternal lipoproteins interact with LDL/VLDL receptors in MVM, resulting in endocytosis and intracellular hydrolysis, which releases FFA. Intracellularly, FFAs are transported bound to FABPs (Duttaroy, 2009). During pregnancy, placental LPL activity increases, and plasma FFA concentrations rise rapidly during the third trimester, thus representing the main class of lipids crossing the placenta (Magnusson-Olsson et al., 2006). In diabetic pregnancy, a wide range of disturbances in lipid metabolism have been described, and maternal lipids seem to be the strongest determinants of fetal growth in GDM newborns (Schaefer-Graf et al., 2011). Both maternal TAGs and FFAs are positively correlated with neonatal weight and fat mass in GDM pregnancy, indicating that maternal dyslipidemia may enhance the availability of lipids to the fetus (Schaefer-Graf et al., 2011). Maternal diet and metabolic status alter placental lipid transport and biology (Lewis et al., 2018). First, the dietary composition may directly change the pool of fatty acids available for uptake by the placenta; however, quality and quantity of fats may alter placental lipid transfer indirectly, such as n-3 fatty acids, which is believed to modulate placental oxidant stress and inflammatory status, and lipid transport (Leghi and Muhlhausler, 2016). Maternal GDM and obesity have been reported to alter placental fatty acid transfer. However, further work is required in understanding the mechanisms of specific placental alterations on lipid transport and metabolism (Lewis et al., 2018).

The critical requirement of these fatty acids in feto-placental unit demands an efficient transport of these LCPUFAs to the fetus by the placenta. The human placenta plays a crucial role in mobilizing the fat stores of maternal adipose and actively concentrating and channeling important LCPUFAs to the fetus *via* multiple mechanisms, including selective uptake by the trophoblast, intracellular metabolic transfer, and selective supply to the fetal circulation. Several membrane proteins are thought to be responsible for the tissue fatty acid uptake of the placenta (Dutta-Roy, 2000b; Duttaroy, 2009). These include the 40-kDa plasma membrane-associated fatty acid-binding protein (FABPpm), the heavily glycosylated 88-kDa fatty acid translocase (FAT), also known as CD36, and a family of 63–70-kDa fatty acid transport proteins (FATP 1–6) (Duttaroy, 2009). FABPpm binds to long-chain fatty acids were isolated and purified from human placental membranes (Duttaroy, 2009). The human placental p-FABPpm, located exclusively on the maternal-facing membranes of the placenta, may be involved in the sequestration of maternal LCPUFAs (Dutta-Roy, 2000b). Radiolabeled fatty acid-binding revealed that p-FABPpm had higher affinities (Kd) and binding capacities (Vmax) for LCPUFAs than other fatty acids. Thus, the presence of p-FABPpm in the placental membrane facing maternal circulation may enforce the uni-directional flow of the LCPUFA from the mother to the fetus. In contrast, FAT and FATPs are present on both microvillous and basal membranes of the human placenta (Dutta-Roy, 2000b). Location of FAT and FATPs on both sides of the bipolar placental trophoblast cells may favor the transport of general FFA pool in both directions, i.e., from the mother to the fetus and vice versa.

FAT/CD36 is heavily glycosylated fatty acid translocase (FAT/CD36), the sequence of which is 85% homologous with that of glycoprotein IV (CD36), is an integral membrane protein (23). This 472-amino-acid (53 kDa) protein is substantially glycosylated (10 predicted N-linked glycosylated sites). Unlike FABPpm, FATP, FAT is a multifunctional protein with several putative ligands, including FFAs, collagen, thrombospondin, and oxidized LDL. FAT/CD36 was demonstrated in the human placenta using both pure trophoblast cells and placental membrane preparations. FAT is present in the placental membranes, microvillous, and basal membranes. Little is known about the regulation of FAT/CD36 function in placental cells, but several lines point toward a translocational mechanism for increasing LCFA uptake by different cells. Caveolin-1 may control FAT/CD36 mediated fatty acid uptake by increasing surface availability.

FATP is the family of integral transmembrane proteins consisting of six (FATP 1–6) isoforms that show different tissue expression patterns. FATP-1 was first identified in human placental membranes. Later other FATP isoforms were detected in the human placenta. Consistent with the role of FATP-1 in fatty acid internalization, a significant portion of FATP-1 is localized at the plasma membrane. FATPs are classified as fatty acid transport proteins because, when overexpressed, they increase the rate of fatty acid internalization, most notably at low concentrations when diffusion may not be sufficient. FATP is suggested to act in concert with fatty acyl-CoA synthetase, an enzyme that prevents efflux of the incorporated fatty acids by their conversion into acyl-CoA derivatives and hence rendering fatty acid uptake uni-directional. These long-chain fatty acyl-CoA esters act both as substrates and intermediates in various intracellular functions. FATP-mediated uptake of long-chain fatty acids was diminished in the face of cellular depletion of ATP. Thus, FATP is likely responsible for the increased uptake of long-chain fatty acids necessary to sustain increased β-oxidation. Therefore, the tissue-selective effects of various PPARs and their ligands produced by FATP and ACS may provide insight into the relationship between FFA uptake, triglyceride synthesis, and β-oxidation of fatty acids. Additionally, FATP-1 possesses ACS activity toward long-chain fatty acids (16–22 carbons), although ACS activity of FATP-1 is very low compared to ACSL1. FATP-1 has one membrane-spanning region and several membrane-associated regions. Since this arrangement does not typically support a channel or transporter, FATP may, therefore, increase the fatty acid internalization by increasing the rate of “flip-flop,” trapping the fatty acids in the inner leaflet of the plasma membrane or activating the fatty acid to its CoA formation. Disruption of the FATP-4 gene in mice demonstrated its essential function in normal mouse development. However, little is known about the specificity of FATPs for different fatty acids (Herrmann et al., 2003).

A transporter major facilitator superfamily domain-containing 2A (MFSD2a) is also reported in the human placenta (Basak et al., 2020). The precise roles of MFSD2a in the placenta is yet to be deciphered. Placental MFSD2a transporter expression decreased and correlated to decreased DHA in cord blood of women with gestational diabetes, indicating its contributiong to materno-fetal DHA transport (Sanchez-Campillo et al., 2020). Not much is known about the cellular localization of MFSD2a in the human placenta yet, although its expression is altered in the placenta of gestational diabetes placenta and preeclampsia (Prieto-Sanchez et al., 2017). Human placental MFSD2a (the major facilitator superfamily domain-containing 2a) also contributes to fetal DHA accumulation by transporting DHA-containing lysophospholipids (Prieto-Sanchez et al., 2017).

PPAR-γ and RXR regulate fatty acid transport in primary human trophoblasts (Schaiff et al., 2005). The incubation of human trophoblast cells with both PPAR-γ and RXR agonists resulted in elevated mRNA expression of FATP-1 and FATP-4 but not FATP-2, -3, and -6 in these cells (Schaiff et al., 2005). Similar results were reported *in vivo* using PPAR-γ agonist that increased the placental expression of FABPpm and FAT/CD36 (Schaiff et al., 2005).

Several FABPs are involved in the fatty acid trafficking of human placental trophoblasts. The complex interactions of these proteins may be essential for active fatty acid transport, metabolism, and gene expression in the placenta. Also, the involvement of several nuclear transcription factors (PPARs, LXR, RXR, and SREBP-1) in the expression of genes responsible for fatty acids uptake, placental trophoblast differentiation, and human chorionic gonadotropin (hCG) production indicates regulatory roles of fatty acid-activated transcriptions factors in placenta biology (Weedon-Fekjaer et al., 2005). The uptake of individual fatty acids was almost equally inhibited by triacsin C (an inhibitor of CoA formation), indicating that the CoA formation step may be involved in the uptake of these fatty acids in the first-trimester human trophoblast cells, HTR8/SVneo (Basak and Duttaroy, 2013). This is again in contrast to what was observed in triacsin C-induced reduction of DHA uptake by the epithelial placental trophoblasts where DHA uptake was inhibited least by triacsin C compared with other fatty acids (Tobin et al., 2009). DHA, by avoiding CoA formation in the last trimester, trophoblasts may allow preferential transport across these cells. In contrast, the first-trimester trophoblast cells are not fatty acid transporting cells like last trimester placental trophoblast cells.

## Maternal Long-Chain Polyunsaturated Fatty Acids and Fetal Brain Development *in Utero*

The maternal, fetal, and neonatal EFA/LCPUFAs status is an important determinant of feto-placental growth and development (Innis, 1991; Uauy et al., 2001). As a structural component, fatty acids are involved in the function of the neuronal membrane. A change in the fatty acid composition of the synaptic membranes can affect neuronal functions in terms of membrane receptors, ion channels, and enzymes and the transmission of intra- and inter-cellular signals generated by fatty acid-derived second messengers. Nervous tissue has the second-highest concentration of fatty acids after adipose tissue, and LCPUFA levels are particularly high in the retina and cerebral cortex. Most LCPUFAs accumulate during brain development at a period of intense cell division, synaptogenesis (Innis, 2008). The importance of DHA during pregnancy in fetal cognitive development and its postnatal spill-over effect has been studied (Anderson et al., 1990; Innis, 2008). The critical role of DHA rests on its participation in maintaining membrane fluidity, impulse propagation, synaptic transmission, and its function as a cytosolic signal-transducing factor for gene expressions during brain development (Anderson et al., 1990; Abdelwahab et al., 2003). In humans, this accumulation starts at the beginning of the trimester and continues until 2 years of age (Clandinin et al., 1980; Innis, 2008).

Maternal LCPUFAs are provided to the offspring *via* placental transfer during gestation and *via* lactation after birth. Consequently, brain LCPUFA in the offspring much depend on maternal PUFA intake (Elias and Innis, 2001). Adequate DHA supply during the perinatal period is essential for optimal CNS development and function. In contrast, dietary n-3 LCPUFA deficiency impairs neuronal plasticity. Animal studies have demonstrated that DHA deficiency during gestation and soon after birth could not be fully corrected later in life (Krabbendam et al., 2007). At 33 weeks, the hypothalamus glycerophospholipids of young pups had significantly reduced DHA compared with controls (that had received ALA for 3 weeks after birth) even after dietary correction with ALA for 30 weeks.

During the third trimester of pregnancy, DHA requirements increase to support fetal growth, particularly of the brain and eyes (Innis, 1991). DHA can account for up to 50% of phospholipid fatty acids in these tissues, suggesting that it is heavily involved in neuronal and visual functions (Simopoulos, 1989; Neuringer, 2000). N-3 LCPUFAs play a well-documented role in the brain development of the fetus and child (Gibson and Makrides, 2000; Uauy and Hoffman, 2000). Premature infants require particular attention, as they have no reserve adipose tissue. These reserves are not generally built up until the third trimester. Premature infants are, therefore, directly dependent on maternal dietary intake of LCPUFAs (Gibson and Makrides, 2000). DHA also showed significant effects on photoreceptor membranes and neurotransmitters involved in the signal transduction, rhodopsin activation, rod and cone development, neuronal dendritic connectivity, and functional maturation of the central nervous system (Neuringer, 2000; Uauy et al., 2001).

DHA deposition in the brain takes place during intrauterine and lactation (Carver et al., 2001; Basak et al., 2021). Accretion of DHA in the fetal brain is the highest in the third trimester of pregnancy (Innis, 2008). DHA incorporation in the neuronal membrane in early life solely depends on placental transfer (Dutta-Roy, 2000a), breastfeeding, and endogenous synthesis of DHA (Cetin et al., 2009; Duttaroy, 2009). Maternal dietary intakes of DHA influence the long-chain fatty acid composition of breast milk and plasma levels of lactating women and their infants (Sherry et al., 2015). A robust linear relationship between maternal DHA level and umbilical cord plasma phospholipid contents was reported (Calder, 2016).

The high-affinity placental plasma membrane fatty acid-binding protein (p-FABPpm) is thought to be involved in maternal transport of DHA to the fetus (Campbell and Dutta-Roy, 1995; Basak et al., 2020). Since the rapid deposition of DHA into the brain occurs during the last trimester and subsequently in lactation, the maternal DHA status must be maintained well during the critical window of brain development. The dietary intake and maternal stores of DHA are the determinants of neonatal blood DHA concentrations at birth (Bourre, 2007). Blood LCPUFA in breastfed infants remain higher than maternal levels for some time postnatally (Koletzko et al., 1989; Jorgensen et al., 2006). Dietary supplementation of DHA to pregnant and nursing mothers dose-dependently increases the DHA level in breast milk, which causes higher tissue accretion of DHA in breastfed infants with improved outcomes of mental performance (Innis, 2005; Oddy et al., 2010; Hibbeln et al., 2019). Comprehensive studies have shown that dietary supplementation during pregnancy with marine oil sources of n-3 LCPUFAs results in increased blood levels of DHA and an associated improvement in visual and cognitive function in infants and children (Table 2).

**TABLE 2:** Effects of maternal long-chain polyunsaturated fatty acids during pregnancy on brain development and growth in infant and children: clinical trials.

Trial	Fatty acid intervention	The major outcome with references
1	DHA 300 mg/d, EPA 42 mg/d, ARA 8.4 mg/d from the third trimester for 12 wk. (*n* = 300)	MRI of newborn infants showed a significant increase in their brain size and correlated with maternal DHA (Ogundipe et al., 2018)
2	DHA 400 mg/d, 16 wk. to delivery (*n* = 271)	Maternal DHA correlates with language and short-term memory development in 5.75 yr. children (Mulder et al., 2018)
3	DHA 600 mg/d, 14.5 wk. to delivery (*n* = 301)	Substantially lowered early pre-term birth and improved visual attention in infancy (Colombo et al., 2019)
4	DHA 120 mg/d and EPA180 mg/d, 20 wk. to post-delivery 1 mo. (*n* = 150)	Improved communication zone of neurodevelopment in 4-mo infants (Ostadrahimi et al., 2018)
5	DHA 600 mg/d, < 20 wk. to delivery (*n* = 350)	Increase in gestational duration and birth size. Reduction in early pre-term delivery (Carlson et al., 2013)
6	DHA 500 mg/d, EPA 150 mg/d, and 5-MTF 400 μg/d from 20 wk. to delivery (*n* = 315)	Higher maternal DHA associated with mental processing score in 6.5 yr children (Campoy et al., 2011). Improved neurological outcome in 5.5 yr children (Escolano-Margarit et al., 2011)
7	DHA 400 mg/d, 18–22 wk. to delivery (*n* = 1,094)	Increased birth size and head circumference at birth is derived from mothers with lower dietary DHA strata (Ramakrishnan et al., 2016). Improved attention in 5 yr. pre-school children (Ramakrishnan et al., 2010)
8	DHA 800 mg/d, < 21 wk. to delivery (*n* = 2,399)	No effects on cognitive development in 1.2 yr. infant (Makrides et al., 2010)
9	DHA 2,200 mg/d and EPA 1,100 mg/d, 20 wk. to delivery (*n* = 98)	Improved hand-eye coordination in 2.5 yr. children (Dunstan et al., 2008)
10	DHA 1,183 mg/d and EPA 803 mg/d, 18 wk. to post-delivery 3 mo. (*n* = 143)	Improved mental processing score in children at 4 and 7 yr. (Helland et al., 2003)

*MRI, Magnetic resonance imaging; mo, Month; yr, Year; wk, Week; d, Day.*

Based on established guidelines, it is emphasized that maternal dietary DHA requirements should be increased during pregnancy and lactation. Precisely, a minimum of 200 mg of DHA per day is recommended during these periods (Van Elswyk and Kuratko, 2009). In both full-term and pre-term, the evidence is compelling that breastfeeding is vital for an infant’s neurodevelopment. Worldwide, mean DHA and ARA levels in breast milk are found 0⋅37 and 0⋅55% of total fatty acids, respectively.

Children’s intelligence quotient (I.Q) was increased by 0.8–1.8 points when their mothers were supplemented with DHA for a more extended period from pregnancy to lactation period and beyond (Cohen et al., 2005; Dunstan et al., 2008). Several prospective studies indicated that breastfed infants had a significant neurocognitive advantage compared with formula-fed infants (Agostoni et al., 1995; Kramer et al., 2008; Oddy et al., 2011), possibly due to the higher incorporation of DHA and ARA in breast milk relative to formula milk. The association between breastfeeding and child IQ concerning the FADS2 genetic profile, specifically in SNP rs174575b, established a genetic variability in the fatty acid metabolism (Caspi et al., 2007). Breastfed infants with rs174575 C-dominant carriers achieved higher scores on standardized IQ tests than non-breastfed C-carriers infants. However, observational data are confounded by the heterogeneous composition of breast milk and environmental factors that influence infant mental development.

Mother supplies both ARA and DHA to the growing and developing brain *via* placenta and breastfeeding. ARA uptake was higher in early trimester trophoblast cells than in EPA and DHA (Basak and Duttaroy, 2013; Basak et al., 2021). ARA may be required in a higher amount to support growth-promoting placental activities and the production of prostaglandins and eicosanoids. In human milk, the amount of ARA typically exceeds the DHA levels (Lauritzen and Carlson, 2011). Milk ARA content is also less varied than DHA, and, unlike DHA, ARA does not seem to be linked to maternal intake (Basak et al., 2021). There has been much discussion in recent years about the proportional requirement of ARA and DHA in infant formula. Studies clearly show the requirement for both ARA and DHA in addition to the essential fatty acids [linoleic acid, 18:2n-6 (LA), and alpha-linolenic acid, 18:3n-3 (ALA)] to support the optimal body and brain growth, and its function. ARA is quantitatively the most predominant LCPUFAs in the brain after the DHA (Contreras et al., 2000). Although DHA’s neuroprotective properties are documented, the roles of ARA in brain development and functions have not been highlighted to a greater extent. Given that ARA and its precursor, LA, contribute significantly to the Western diet and its pleiotropic biological effects and interactions with DHA make this n-6 LCPUFA a crucial modifiable factor in brain development and preventive strategies of brain diseases. ARA corresponds to around 20% of neuronal fatty acids and is mainly esterified in membrane phospholipids.

Several studies have suggested that the structure-function and metabolism of the brain depend on levels of ARA, DHA and interactions of their metabolites (Contreras and Rapoport, 2002). ARA must either be consumed in the diet or synthesized from its precursor LA in the liver. The brain contains relatively low LA levels, and its conversion into ARA is minimum in the brain. Thus, the brain depends on a steady supply of ARA from the circulation. Although lipoproteins and lysophospholipids of plasma may contribute to brain ARA levels, their quantitative contribution is unknown. Plasma unesterified ARA deposits in the brain at the rate of 17.8 mg/day in the whole brain of adult humans. Upon its entry into the brain, ARA is activated by a long-chain acyl-CoA synthetase and can be esterified into the sn-2 position of phospholipids. During neurotransmission, the brain ARA cascade is initiated when it is released from synaptic membrane phospholipid by neuroreceptor-initiated activation of cPLA_2_. PLA_2_ is activated by dopaminergic, cholinergic, glutamatergic, and serotonergic stimulation *via* G-proteins or calcium (Sun et al., 2010). Several PLA_2_ are activated *via* serotonergic (5-hydroxytryptaminergic), glutamatergic, dopaminergic, and cholinergic receptors (Bazinet, 2009; Sun et al., 2010). Usually, calcium-dependent cytosolic PLA_2_ (cPLA_2_) resides at the postsynaptic terminals, selective for releasing ARA, whereas calcium-independent PLA_2_ is believed to remove the DHA sn-2 position phospholipids [13,14]. Upon its release, a portion of the unesterified ARA is converted to prostaglandins, leukotrienes, and lipoxins, a portion oxidized *via* β-oxidation, and the remainder (approx. 97% under basal conditions) is activated by ACSL and ultimately recycled and re-esterified into the sn-2 position of phospholipids (Rapoport, 2008). An additional ARA is released by activated cytokine and glutamatergic N-methyl-d-aspartate receptors in conditions such as neuroinflammation and excitotoxicity. Although ARA and its derivatives relay signals are not entirely understood, they regulate cellular growth and differentiation, blood flow, neuroinflammation, excitotoxicity, the sleep/wake cycle, and neurogenesis (Duncan and Bazinet, 2010).

ARA itself is also directly involved in synaptic functions. The level of intracellular free ARA and the balance between the releasing and incorporating enzymes in membrane phospholipids may play critical roles in neuroinflammation and synaptic dysfunction. The substrate binding and trafficking of ARA and DHA are mediated by FABPs (Mallick et al., 2021). For example, FABP5 and FABP7 are more selective for DHA, whereas FABP3 binds ARA with much higher affinity (Boneva et al., 2011; Pan et al., 2015). Furthermore, regarding fatty acid metabolizing enzymes, DHA is preferentially used as a substrate by ACSL6 (Marszalek et al., 2005) and calcium-independent group VI PLA_2_ (Green et al., 2008), whereas ACSL4 and group IV cPLA_2_ used ARA as preferred substrate.

Both ARA and DHA are sources of phospholipids in the membrane fluidity of synapse that controls the functions of receptors, transporters, and membrane-bound proteins. ARA and DHA are required to replenish brain injury, vascular regulation, and brain development, and maturity in pre-term babies. ARA and its metabolites are needed to support endothelial cells during brain injury. In contrast, DHA and its metabolites are required to support membrane fluidity for the receptors, linear growth and network of neuronal cells. Neuroprotective actions of DHA in pre-term are manifested with anti-inflammatory initiation and resolutions by its own and its signaling lipid mediators in particular. ARA and DHA produced different sets of pro-resolving lipid bioactives. Lipoxins are derived from ARA, while resolving and maresins are derived from EPA and DHA. LipoxinA4 (LXA4) receptor staining during brain injury indicates that LXA4 lowers neuroinflammation and brain edema (Luo et al., 2013; Basak et al., 2020). The anti-inflammatory effects of LXA4 acts as an endogenous allosteric modulator of the cannabinoid receptor (Pamplona et al., 2012). Thus, in contrast to PGE_2_, which shows pro-inflammatory actions, lipoxins offer an inflammation resolution process (Basak et al., 2020).

## Conclusion

The essentiality of both DHA and ARA in feto-placental development is corroborated by the fact that the mother is required to supply both preferentially during the critical window of brain development *via* the placenta and breastfeeding postnatally. Both ARA and DHA are necessary for fetal neurodevelopment, and a deficiency may compromise the optimum growth of the babies. Moreover, early placental angiogenesis is critical for establishing the placental size and vascularization and, thus, average fetal growth and development. LCPUFAs favor placental growth by increasing angiogenesis in the first-trimester placenta cells. The dietary fatty acids stimulated the expression of major proangiogenic growth factors such as VEGF and ANGPTL4, FABP4, FABP3, which modulate angiogenesis directly. The human placental trophoblasts play a crucial role in mobilizing the maternal fatty acids and channeling important LCPUFAs to the fetus *via* multiple mechanisms, including their selective uptake by the trophoblast, intracellular metabolic trafficking, and selective export to the fetal circulation. Among the fatty acid-binding/transport proteins, p-FABPpm, located exclusively on the maternal-facing membranes of the placenta, may be involved in the sequestration of maternal LCPUFAs by the placenta. A better understanding of the physiology of fatty acid transport of the fetoplacental unit is needed in order to secure optimum fetoplacental growth and development.

## Author Contributions

AD: writing—original draft preparation and writing—review and editing. SB: contributed to editing and revision of the work. Both have approved the final version for submission.

## Conflict of Interest

The authors declare that the research was conducted in the absence of any commercial or financial relationships that could be construed as a potential conflict of interest.

## Publisher’s Note

All claims expressed in this article are solely those of the authors and do not necessarily represent those of their affiliated organizations, or those of the publisher, the editors and the reviewers. Any product that may be evaluated in this article, or claim that may be made by its manufacturer, is not guaranteed or endorsed by the publisher.

## Abbreviations

ARA: Arachidonic acid,20:4n-6
ALA: α-linolenic acids 18:3n-3
COX: Cyclooxygenase
DHA: Docosahexaenoic acid,22:6n-3
EPA: Eicosapentaenoic acid, 20:5n-3
LCPUFAs: Long-chain polyunsaturated fatty acids
EFA: Essential fatty acids
LA: Linoleic acid, 18:2n-6
FABPs: Fatty acid-binding proteins
FAT: fatty acid translocase
FATP: Fatty acid transporter protein
FABPpm: plasma membrane-fatty acid-binding protein
LOX: Lipoxygenase
VEGF: Vascular endothelial growth factor
ANGPTL4: Angiopoietin-like protein 4.
